# Environmentally related gender health risks: findings from citizen science cross-sectional study

**DOI:** 10.1186/s12889-022-13824-3

**Published:** 2022-07-27

**Authors:** Regina Grazuleviciene, Sandra Andrusaityte, Aurimas Rapalavicius, Audrius Dėdelė

**Affiliations:** 1 Department of Environmental Science, Vytautas Magnus University, 44248 Kaunas, Lithuania; 2 Department of Family Medicine, Lithuanian University of Health Sciences, 48005 Kaunas, Lithuania

**Keywords:** Urban built environment, Air pollution, Social environment, Neighborhood quality perception, Gender health, Citizen science

## Abstract

**Background:**

Public engagement in the research of environmental epidemiological problems is becoming an important measure to empower citizens to identify the local environmental and health problems and to explain different environmental exposures affect estimates for males and females. This HORIZON2020 CitieS-Health Kaunas Pilot study examines the relationship between urban built and social environment, health behaviors, and health in men and women.

**Methods:**

This cross-sectional study included 1086 18–74-year-old participants residing in 11 districts of Kaunas city, Lithuania. Using GIS, we measured traffic flow, noise, NO2, PM2.5, PM10, and greenness NDVI for the participants’ home addresses, determined participants’ perceptions of environmental quality, linked this information with personal sociodemographic data, and used multivariate logistic regression to assess the associations with health issues (physician-diagnosed chronic disease and self-rated general health) in men and women.

**Results:**

Men and women similar rated the quality of the neighborhood environment, except for air pollution and satisfaction with the public transport in the district. The traffic-related health associations were stronger for women than for men. The prevalence of poor health increased with the increasing age of men and women, yet no significant differences between gender health risks were found in the total sample. Perceived air pollution, irregular visits to green space, and chronic diseases were consistently associated with poor health risks in men and women, yet part-time jobs and low income had a higher impact on women’s poor health.

**Conclusions:**

Quality of the built neighborhood, air pollution, irregular visits to the green space, and chronic disease had a joint effect on the magnitude of the prevalence of poor health in men and women. Our results suggest that decreasing air pollution and improving the urban built neighborhood supporting citizens’ physical activity in green spaces, might reduce health risks for all.

**Supplementary Information:**

The online version contains supplementary material available at 10.1186/s12889-022-13824-3.

## Background

Sustainable cities and communities’ development tackles public concerns of sustainable environment and citizens’ well-being and requires a greater awareness of communities on issues of the local environment and health, and purposeful activity to reduce inequalities. The European Commission presented a policy on how the Member States can reach the 17 Sustainable Development Goals (SDG), among them SDG 5 Gender Equality, as an important constituent of reducing poor health outcomes. Some activities are devoted to ensuring healthy lives and to promoting well-being for all, women empowerment, reducing inequalities in health status, and promoting social inclusion [1]. Women’s and men’s engagement in the collaborative study could make important contributions to societal transformations and could exert pressure on politicians to solve environmental problems and health disparities [2, 3]. So far, the available literature on gender differences remains conceptually groundless about the multidimensional gender concepts for quantitative environmental health research [4]. Recent research has shown positive links between satisfaction with environmental conditions, well-being, and physical health [5, 6]. The perceived quality of the outdoor environment might encourage and enhance or discourage the physical activity of women in green spaces and might be promising in studying mechanisms underlying female health [7]. While integrating gender theoretical concepts into environmental health research, have the potential to improve the validity of research and, thus, support the promotion of measures for health equity in society.

There is some evidence of gender-specific health risks including perceived overall health or wellbeing due to biological differences [8, 9], differences in unfavorable socioeconomic and environmental stressors [10], and personal characteristics [11, 12]. Even though women have a longer life expectancy than men, they report poorer general health [13]. This phenomenon can partly be explained by biological differences between males and females [14–17], by differences in SES [18–21], by behavioral factors and the psychosocial environment [22, 23], or by differences in response to exposure to environmental stressors [24, 25]. There are some data that citizens’ physical health and well-being depend on the urban built neighborhood, which comprises both social and physical environments including green spaces [26, 27]. It is assumed that green space is an important component of the health and well-being in urban areas [28]. Potential pathways linking green space to health comprise reducing exposure to air pollution, noise, physiological stress, and encouraging physical activity [29]. Both physiological and psychological responses to greenness may differ between females and males [30, 31], for people of lower socioeconomic status [32], and residents of urbanized settings [33]. Inconsistency in health disparities may also depend on the traffic-related environmental pollution levels [34, 35] significant social inequalities in environmental exposures, which exist between and within countries, as well as within communities [10, 18, 36]. The current literature indicates that in most environmental health and well-being studies gender health effects varied, with no consistent findings for both males and females. These studies currently are focus on physical or social neighbourhood exposure [37], yet only some have focused on gender health disparities [38–42] or an understanding of how the living environment influences physical activity for health promotion and underlying motivational processes [43].

Advancing health equity studies by environmental epidemiological research could provide the possibility to an expanded understanding of disparities in gender health issues, to gain new data on gender concepts for quantitative environmental health research and to deliver measures for implementation health equity and well-being for all [35, 44–46].

So far, only very few studies have examined the relationship between community-level environmental stressors, personal characteristics, and social determinants of genders health [24, 47].

**Fig. 1:**
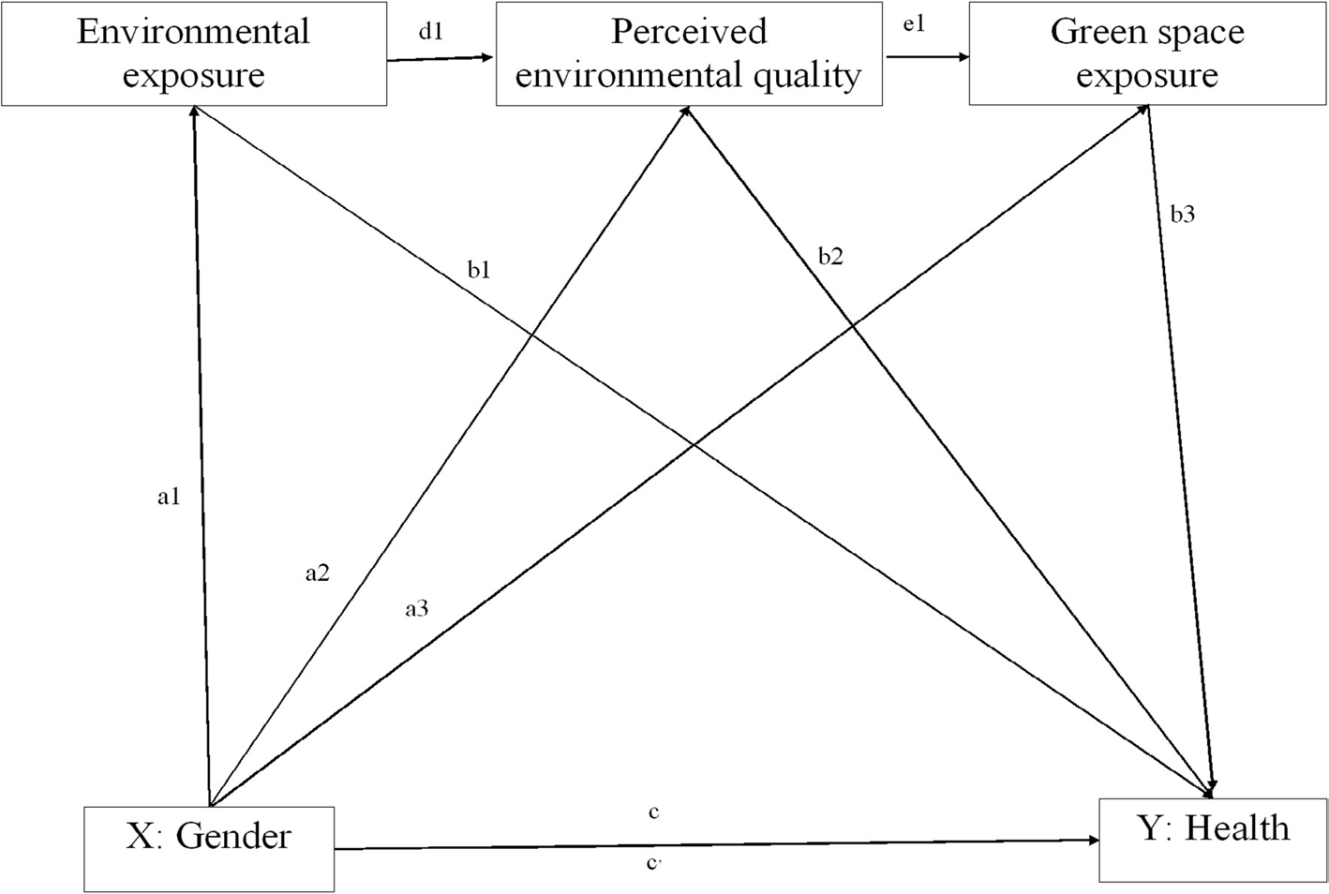
Conceptual framework showing hypothesized pathways linking gender to health outcomes. The arrows represent hypothetical patterns of influence

Based on previous studies, we present hypothetical pathways linking gender to health outcomes (Fig. 1) in which explore the associations between the objectively measured and subjectively measured (perceived) quality of the neighborhood, green space exposure (physical activity in green space), sociodemographic factors, and men’s and women’s health. The study has been initiated as the Kaunas Pilot study of the Horizon 2020 proposal Citizen Science for Urban Environment and Health [48]. The previously published findings showed that the poor quality of the neighborhood and individual-level stressors had an effect on a higher prevalence of health problems at the city district level [49]. In this study, we have measured objective physical exposure indicators (traffic flows, noise, air pollution (NO2, PM2.5, PM10), and greenness level (NDVI), measured subjective (perceived) residential environmental quality indicators (the infrastructure in the residence neighborhood, social environment, safety), personal sociodemographic data, and both hard health outcomes (physician-diagnosed chronic disease, hypertension, diabetes, obesity, allergy) and self-rated general health. Self-rated health is treated as valid predictor of morbidity and mortality [50, 51] and only slight differences in validity between women and men have been observed [52].

Building on the participants’ concerns for the environmental health and well-being, this study had two objectives: 1) to estimate if there is a difference in the perception of the neighborhood quality and risk factors for poor self-rated health in men and women; and 2) to test the hypothesis that the built and social environment may have a different impact on men’s and women’s general health. The joint research of different urban built neighborhood and social environmental factors in relation to health has a potential of presenting evidence on health effects of modifiable environment-related and behavioral factors for interventions to improve all citizens’ health and well-being.

## Materials and methods

### Study design

The participants in this collaborative study were enrolled from 2019 to 2020. During the first stage of the engagement, 580 18–75-year-old participants were enrolled using face-to-face interviews. During the second stage (COVID-19 pandemic), 506 45–64-year-old participants were randomly selected using voting lists and were engaged in the study via an internet survey. The study sample consisted of 1086 18–75-year-old participants permanently residing in 11 districts of Kaunas city, Lithuania.

The city covers 15,700 ha and includes 8 city parks (areas larger than 1 ha), with 65% of land covered with trees. All Kaunas city parks are open to the public and are located amidst residential homes or establishments and near to the public transport lines. The city parks offer some recreation opportunities (e.g., walking, jogging, rollerblading, physical training, or resting on the bench).

A detailed description of the methods of the participants’ enrolment as well as the description of the collaborative study have been provided previously [53, 54]. The study was conducted in accordance with the Declaration of Helsinki [55] and were approved by the Kaunas Regional Committee for Biomedical Research Ethics (BE-2-51. 2019-06-10). All participants filled out the formalized questionnaire which had closed-ended and open-ended questions for the clarification of the study participants’ opinion, suggestions, and concerns about local ecological issues and health. Collected survey information comprised on the quality of the built neighborhood, residence history, socio-demographic and socioeconomic factors, physician-diagnosed chronic diseases, health behavior, and self-rated general health. We conducted a cross-sectional study and analyzed associations between environmental issues and health outcomes in men and women. All analyses were conducted stratified by gender.

### Measurements

#### Environmental-related indicators

Participants’ environmental exposures were estimated by the ArcGIS 10.4 software. Residence on the street with more than 10,000 cars per day was treated as high exposure to traffic emissions. The average annual value of nitrogen dioxide (NO2) and particulate matter (PM2.5 and PM10) for each district was estimated by LUR model (2014) and noise level (Lden) was estimated using Strategic noise map of Kaunas city (2012). The assessment of residential greenness was based on a NDVI that was derived from Landsat 7 Enhanced Thematic Mapper Plus (ETM+) data at 30 m × 30 m resolution [56]. The maps of NDVI were generated using the image that was obtained in 2014 summer season, with an average cloudiness of < 10%. The NDVI index for each district was calculated using ArcGIS 10.4 software.

Perceived residential environmental quality indicators were estimated by using a seven-point Likert rating scale ranging from 1 (strongly disagree) to 7 (strongly agree). We asked the participants to rate statements about their current residential environment as follows: “How would you rate your neighborhood, built environment, and social environment on a scale from 1 (strongly disagree) to 7 (strongly agree) using below presented statements?”

The statements on the infrastructure in the residence neighborhood: the public transport in the district meets my needs; I am satisfied with pathways and cycling routes; there are opportunities for walking to reach the city’s green spaces or parks.

The statements on social cohesion and safety: I can take part in decision-making to improve the environment in which I live; I feel safe in my area; during the last 6 months, I have felt stress, tension, or anxiety. The statements on perceived environmental quality: the air pollution in my place of residence cause problems; the noise in my place of residence hinders my sleep and/or work at home. Higher scores indicated better neighborhood conditions.

#### Health outcomes

The participants’ health status was assessed by the presence or absence of physician-diagnosed chronic disease, obesity, hypertension, systolic and diastolic blood pressure, and the body mass index (BMI) calculated using the measures of body weight and body height. Self-rated general health was measured by asking the participants to answer the question “How would you rate your overall health status at present on a scale from 1 (strongly disagree) to 5 (strongly agree)?” The self-rated general health using a five-point Likert rating scale is used in the international studies [57]. We validated the study participants’ reporting of physician-diagnosed hypertension using responses on blood pressure readings. To ensure that the data are comparable, we compared the prevalence of self-reported physician-diagnosed hypertension with the professionally collected data of a random sample of the inhabitants of Kaunas city [11].

#### Socioeconomic factors

Individual-level predictors of the SES were assessed by evaluating the participants’ education level, situation at work, and income. The education level was ascertained in years and in the analysis, we used a binary operationalization lower education/higher education (university) group. The situation at work was ranked as full-time/part-time, and the monthly net income was also ranked as low (less than 400 Euros)/higher (400 Euros or more).

#### Behavioral factors

Smoking status was self-reported as nonsmoker/current smoker. The participants presented information on physical activity during leisure time by answering the following question: “During the last week, what was the mean time per day you spent outdoors by fast walking, bicycling, or gardening?” The measure of physical activity was adapted from the publicized international studies [57]. We validated the consistency of the answers by comparing the above-mentioned time with time spent in a park and with the professionally collected data of a random sample of Kaunas citizens [58]. In this study, the recommended duration of physical activity was defined by the international guidelines [59], i.e., at least 150 min/week of moderate-intensity physical activity outdoors. Physical activity during analysis was classified into two groups: recommended - at least 150 min/week of moderate-intensity physical activity, and fewer min/week spent outdoors.

### Analysis

#### Descriptive statistics

To examine risk factors for poor self-rated health in men and women, we first tabulated frequency distributions of the characteristics. The baseline characteristics were examined using the chi-squared test. The mean environmental perception score was used to evaluate the situation in the residential district. In tables we reported mean values and standard deviations and choose a *p*-value < 0.05 as the significance level. Second, we estimated different factors influencing men’s and women’s health and well-being. The qualitative characteristics of the groups were compared using Fisher’s exact tests.

#### Modeling of main effects

Thirdly, we applied multivariate logistic regression models to assess the impact of built and social environment variables and green space exposure on men’s and women’s health and health disparities. The relationship between the variables was estimated as odds ratios (OR) and their 95% confidence intervals (CI). In the stratified by gender multivariate logistic regression models, we applied higher than 0.05 *p*-value thresholds (such as < 0.2) for the inclusion of predictor variables from bivariate statistics in order to prevent the exclusion of relevant factors [60]. For this reason, we also retained the variables that changed the adjusted odds ratios (aOR) by 10% or more for inclusion in the multivariate logistic regression analysis. In the statistical analysis, we dichotomized personal data and used mean values of environmental perception scores as cut points for an easier interpretation of logistic regression estimates. The following covariates were included in the models: sex (men, women), family status (married, other), smoking status (no, yes), education level (lower education status, university), age (continuous), situation at work (full-time, part-time), monthly income (< 400€, 400€ and more), NDVI. We conducted a stratified logistic regression analysis to explore the role perception of air pollution in the neighborhood as a factor modifying the effects of traffic flow on self-rated health in participants. Statistical analyses were performed using SPSS version 25.0 package (IBM Corporation, New York, NY, USA).

## Results

### Descriptive statistics for men and women

The study included 498 men and 588 women aged 18–74 years. Gender differences were found in age, income, situation at work, smoking, body mass index, and diastolic blood pressure (Supplementary material, Table S1). Men more often were full-time employees (*p* < 0.001) with a higher monthly net income (*p* = 0.03). They also smoked more often than women. There were no significant differences in the men’s and women’s traffic-related environmental exposures. Physical activity was low in both men and women, mostly not reaching the overall recommended duration of physical activity (at least 150 min/week of moderate-intensity physical activity outdoors). The mean prevalence of poor self-rated health among 18–74 years men and women were similar (14.1 and 16.5%, respectively, *p* = 0.311).

**Fig. 2:**
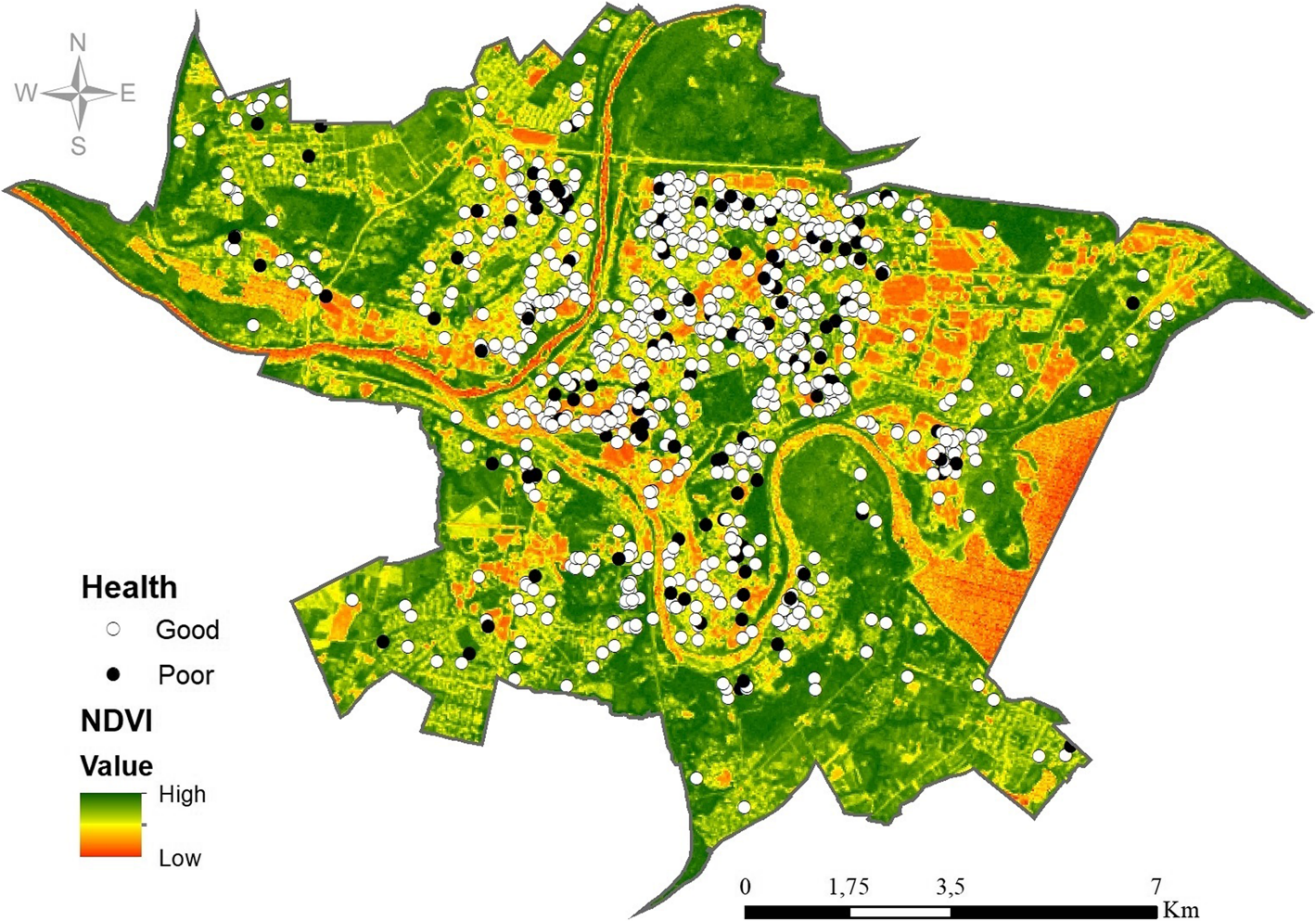
Spatial distribution of exposure to greenness (by NDVI) and the prevalence of self-rated poor health of the study participants in Kaunas city

We explored spatial patterning in greenness NDVI and the prevalence of self-rated health status (good/poor) of the study participants at the Kaunas city level (Fig. 2).

The male/female proportion across the participants of different districts were similar.

### Risk factors for poor self-rated health in men and women

To estimate risk factors influencing poor health, we performed an analysis by women and men individual characteristics and the self-rated health status (Table 1).

**Table 1:** Risk factors for women and men self-rated poor health

Personal characteristics	Women		***p***	Men		***p***
	Good health, N (%)	Poor health, N (%)		Good health, N (%)	Poor health, N (%)	
Age groups			*< 0.001‡*			*< 0.001‡*
18–44	173 (92.5)	14 (7.5)		164 (94.8)	9 (5.2)	
45–64	295 (82.6)	62 (17.4)		253 (81.6)	57 (18.4)	
> = 65	23 (52.3)	21 (47.7)		10 (71.4)	4 (28.6)	
Family status			*0.737‡*			*1.000‡*
Married	275 (84.1)	52 (15.9)		253 (85.8)	42 (14.2)	
Other	216 (82.8)	45 (17.2)		174 (86.1)	28 (13.9)	
Educational status			*0.738‡*			*0.197‡*
Lower	217 (82.8)	45 (17.2)		206 (83.7)	40 (16.3)	
University	274 (84.0)	52 (16.0)		221 (88.0)	30 (12.0)	
Situation at work			*< 0.001‡*			*< 0.771‡*
Full-time	316 (87.5)	45 (12.5)		314 (85.6)	53 (14.4)	
Part-time	174 (77.0)	52 (23.0)		112 (86.8)	17 (13.2)	
Monthly net income			*0.002‡*			*0.708‡*
Less than 400 €	78 (72.9)	29 (27.1)		58 (87.9)	8 (12.1)	
More than 400 €	413 (85.9)	68 (14.1)		369 (85.6)	62 (14.4)	
Smoking			*0.492‡*			*0.325‡*
No	383 (83.1)	78 (16.9)		295 (84.8)	53 (15.2)	
Yes	106 (86.2)	17 (13.8)		132 (88.6)	17 (11.4)	
Perceived air pollution problems			*< 0.001‡*			*< 0.029‡*
Yes	244 (77.2)	72 (22.8)		201 (82.4)	43 (17.6)	
No	247 (90.8)	25 (9.2)		226 (89.3)	27 (10.7)	
Visits to green space			*0.003‡*			*0.041‡*
Irregular	276 (79.8)	70 (20.2)		176 (82.2)	38 (17.8)	
Regular	215 (88.8)	27 (11.2)		251 (88.7)	32 (11.3)	
Recommended physical activity			*0.636‡*			*0.213‡*
No	419 (83.1)	85 (16.9)		357 (85.0)	63 (15.0)	
Yes	72 (85.7)	12 (14.3)		70 (90.9)	7 (9.1)	
Chronic disease			*< 0.001‡*			*< 0.001‡*
No	370 (93.0)	28 (7.0)		321 (91.7)	29 (8.3)	
Yes	121 (63.7)	69 (36.3)		106 (72.1)	41 (27.9)	
Hypertension			*< 0.001‡*			*< 0.001‡*
No	378 (89.4)	45 (10.6)		315 (89.5)	37 (10.5)	
Yes	113 (68.5)	52 (31.5)		112 (77.2)	33 (22.8)	
Diabetes			*< 0.001‡*			*< 0.001‡*
No	480 (85.3)	83 (14.7)		418 (87.1)	62 (12.9)	
Yes	11 (44.0)	14 (56.0)		9 (52.9)	8 (47.1)	
Allergies			*0.011‡*			*0.144‡*
No	460 (84.7)	83 (15.3)		408 (86.4)	64 (13.6)	
Yes	31 (68.9)	14 (31.1)		19 (76.0)	6 (24.0)	
Obesity			*0.003‡*			*0.003‡*
BMI < 30	432 (85.0)	76 (15.0)		380 (88.4)	50 (11.6)	
BMI > =30	49 (70.0)	21 (30.0)		42 (67.7)	20 (32.3)	
Traffic 10,000 cars/day			*0.537‡*			*0.571‡*
No	356 (84.2)	67 (15.8)		300 (85.2)	52 (14.8)	
Yes	135 (81.8)	30 (18.2)		126 (87.5)	18 (12.5)	

‡ *p* value of the chi-squared test

Among women of good health and poor health groups’ significant differences were found in individual-level demographic variables, socio-economic variables, and prevalence of chronic diseases. However, no differences were found in objectively measured environmental exposures. There were no differences between the groups in health-related behavior variables – the prevalence of smoking and recommended physical activity outdoors. The factors significantly associated with poor self-rated health risk among women were age, situation at work, income, and physician-diagnosed chronic diseases. The factors significantly associated with poor self-rated health risk among men were age, and physician-diagnosed chronic diseases.

### Gender differences in the perception of the neighborhood quality

We performed an analysis to study whether the perceptions of the environmental quality of the living district and social well-being depended on gender. The mean environmental perception score was used to evaluate the situation in the residential district (Table 2). Higher scores indicated better neighborhood quality and social well-being.

**Table 2:** Mean ratings of the perceptions of neighbourhood quality and social well-being by gender

Neighborhood quality and social well-being	Men mean (SE)	Women mean (SE)	***p***
Satisfaction with built environment			
Satisfaction with public transport in the district	5.11 (0.090)	5.38 (0.077)	*0.022*
Satisfaction with pathways and cycling routes	4.89 (0.095)	5.02 (0.086)	*0.292*
Opportunities for walking to reach park	5.23 (0.093)	5.29 (0.087)	*0.636*
Environmental exposure			
Perceived of air pollution in place of residence	4.10 (0.138)	3.70 (0.120)	*0.030*
Perceived noise at home	4.72 (0.139)	4.78 (0.123)	*0.756*
Regular parks visit Social cohesion	4.57 (0.096)	4.63 (0.087)	0.629
Feeling of safety in the place of residence	5.22 (0.078)	5.07 (0.076)	*0.158*
Possibility take part in decision-making	3.23 (0.096)	3.48 (0.093)	*0.064*
Stress or anxiety during the last 6 months	4.23 (0.087)	4.14 (0.081)	*0.406*

All neighborhood perception scores ranged from 1 to 7: 1 = strongly disagree, and 7 = strongly agree. Higher scores indicate better neighborhood conditions

The participants of both groups (men and women) similarly acknowledged that there were good opportunities for walking to reach the city’s green spaces or parks (*p* = 0.636) and were satisfied with pathways and cycling routes. The participants of both groups highly rated the public transport in the district, indicating that it met their needs, yet women mean rating was higher than men (*p* = 0.022). Male participants less worried about problems caused by air pollution. However, both men and women similarly rated the impact of noise in their place of the residence, stress, tension, or anxiety felt during the last 6 months, or the feeling of safety in the place of residence and possibility to take part in decision-making. These results show some differences between gender in perception of air pollution and well-being.

Subsequently, using logistic regression models, we studied associations between gender, age groups and poor self-rated health (Supplementary material, Table S2). The table present results a sensitivity analysis of univariate models and multivariate logistic regression models adjusted for co-variates. An increase pattern of adjusted odds ratios for poor health varied by gender age groups. However, no significant differences between gender health risk of 18–75 participants were found after full adjustment for age, educational status, family status, situation at work, monthly net income, NDVI, and smoking status. The women’s poor health odds ratios were 1.05, 95% CI 0.74–1.49, *p* = 0.38. The results showed that in this population sample, women gender per se was not risk factor for poor self-rated health.

### Impact of environmental and demographic factors on women’s and men’s poor self-rated health

Seeking to test the hypothesis that the built and social environment may have a different impact on men’s and women’s general health, we conducted an analysis of factors associated with poor self-rated health. Using the univariate and multivariate (adjusted) logistic regression models, we evaluated the associations among individual-level factors, built and social environment factors, and the risk of poor health among men and women, controlling the influence of the possible confounding variables, and determined the strength of the association as odds ratios (Table 3).

**Table 3:** Associations between environmental, sociodemographic, individual-level characteristics and men and women poor self-rated health

Characteristics	Men poor health		Women poor health	
	Univariate OR (95% CI)	aOR (95% CI)	Univariate OR (95%CI)	aOR (95% CI)
Educational status				
University	1(referent)	1(referent)	1(referent)	1(referent)
Lower†	1.43 (0.86–2.38)	1.62 (0.96–2.74)	1.09 (0.71–1.69)	1.08 (0.69–1.72)
Lower††		1.25 (0.71–2.21)		0.84 (0.49–1.43)
Family status				
Married	1(referent)	1(referent)	1(referent)	1(referent)
Other†	0.97 (0.58–1.62)	1.12 (0.66–1.89)	1.10 (0.71–1.71)	1.10 (0.69–1.73)
Other ††		0.98 (0.54–1.87)		0.82 (0.48–1.38)
Situation at work				
Full-time	1(referent)	1(referent)	1(referent)	1(referent)
Part-time†	0.90 (0.50–1.62)	0.75 (0.40–1.40)	2.10* (1.35–3.26)	1.72* (1.08–2.74)
Part-time††		0.94 (0.62–2.14)		1.64 (0.90–2.98)
Monthly net income				
< 400 €††	0.82 (0.37–1.80)	0.51 (0.22–1.18)	2.26* (1.37–3.71)	1.68 (0.97–2.86)
< 400 €†		0.54 (0.19–1.57)		1.23 (0.62–2.45)
≥ 400 €	1(referent)	1(referent)	1(referent)	1(referent)
NDVI				
< mean††	0.78 (0.35–1.73)	0.79 (0.46–1.33)	0.96 (0.52–1.75)	0.89 (0.55–1.43)
< mean†		0.75 (0.43–1.32)		1.08 (0.64–1.79)
> mean	1(referent)	1(referent)	1(referent)	1(referent)
Perceived air pollution				
Yes††	1.79* (1.07–3.00)	1.76* (1.04–2.98)	2.92* (1.79–4.75)	3.30* (1.98–5.51)
Yes†		1.66 (0.95–2.92)		3.12* (1.80–5.39)
No	1(referent)	1(referent)	1(referent)	1(referent)
Regular visits to green spaces				
No††	1.69* (1.02–2.82)	1.67* (1.22–3.63)	1.63* (1.05–2.52)	1.81* (1.14–2.88)
No†		1.66 (0.93–2.92)		1.85* (1.11–3.08)
Yes	1(referent)	1(referent)	1(referent)	1(referent)
Recommended physical activity				
No††	1.77 (0.78–4.01)	1.78 (0.77–4.08)	1.22 (0.63–2.34)	1.39 (0.70–2.73)
No†		1.67 (0.69–4.02)		1.02 (0.48–2.14)
Yes	1(referent)	1(referent)	1(referent)	1(referent)
Allergies				
No	1(referent)	1(referent)	1(referent)	1(referent)
Yes†	2.01 (0.78–5.23)	1.85 (0.69–4.94)	2.50* (1.28–4.91)	2.68* (1.31–5.49)
Yes††		1.91 (0.72–5.02)		2.68* (1.34–5.35)
Obesity				
BMI < 30	1(referent)	1(referent)	1(referent)	1(referent)
BMI > =30†	3.62* (1.97–6.65)	3.16* (1.69–5.92)	2.44*(1.38–4.29)	1.64 (0.91–2.99)
BMI > =30††		3.82* (1.91–7.63)		1.87 (0.95–3.71)
Chronic disease				
No	1(referent)	1(referent)	1(referent)	1(referent)
Yes†	4.28* (2.54–7.23)	3.41* (1.96–5.91)	7.54*(4.64–12.24)	6.11* (3.65–10.23)
Yes††		3.06* (1.57–5.96)		6.40* (3.41–12.00)
Hypertension				
No	1(referent)	1(referent)	1(referent)	1(referent)
Yes†	2.51* (1.50–4.20)	2.17* (1.28–3.68)	3.87* (2.46–6.07)	2.89* (1.78–4.68)
Yes††		2.55* (1.51–4.31)		3.56* (2.25–5.65)

*p < 0.05; OR univariate odds ratios; †aOR adjusted odds ratios for: age (continuous) and smoking status; ††adjusted OR additionally for: educational level, family status, situation at work, monthly income, and NDVI

In men and women groups, after full adjustment for co-variables, SES was not consistently associated with poor self-rated health. Associations between perceived air pollution in the place of residence and poor health were stronger among women than among women. Low physical activity (not reaching recommended level) tended to increase in the risk of poor self-rated health in men and women. However, irregular visits to green space were consistently associated with increased health risk in men and women. Our findings show that physician-diagnosed chronic diseases and hypertension in men and women had the strongest impact on the association with poor self-rated health. Allergies were other important factor that was significantly associated with women’s health, while obesity had higher impact on men’s poor self-rated health. These data provide evidence that individual-level factors and the perceived quality of the neighborhood had different effect on the risk of poor self-rated health in men and women. However, we cannot rule out the possibility that even though we controlled associations for the possible confounding variables, such as gender, age, smoking status, family status, situation at work, monthly income, and NDVI, residual confounding by personal characteristics may have impact on the gender differences in poor health risk.

Then, using a stratified logistic regression analysis, we studied modifying effects of perceived air pollution on the associations between traffic flow and the risk of poor self-rated health in men and women (Table 4). Then, using a stratified logistic regression analysis, we studied modifying effects of perceived air pollution on the associations between traffic flow and the risk of poor self-rated health in men and women. The univariate and multivariate logistic regression models showed that the high traffic flow among the participants with perceived air pollution does not cause health problems, tended to increase in the odds ratios of poor self-rated health in total sample of men and women, and in women only. However, high traffic flow among the participants with perceived air pollution cause health problems, was associated with a significant increase in the risk of poor self-rated health in the unadjusted (OR 2.12, 95% CI 1.29–3.50) and the adjusted (aOR 2.14, 95% CI 1.29–3.55) models. Similar pattern was found in women. These associations were robust to a sensitivity analysis after adjustment for age, educational status, family status, situation at work, monthly net income, NDVI (continuous), and smoking status. In men the traffic flow and perceived air pollution impact in the risk of poor self-rated health was non-significant. The results revealed that both traffic flow > 10,000 cars/day and perceived air pollution contributed to the disparities in the risk of poor health in men and women. Our findings suggest that the perception of air pollution in their place of the residence causing health problems modified the effects of traffic flow on self-rated poor health in women and significantly increase in the risk of poor self-rated health in this group.

**Table 4:** The relationships between traffic flow, perception of air pollution, and the risk of poor self-rated health in men and women (stratified analysis)

Traffic and air pollution	Univariate OR (95% CI)	Adjusted aOR‡ (95% CI)
Men and women poor health		
Traffic < 10,000 cars/day and &Air pollution does not cause problems	Referent group	Referent group
Traffic > 10,000 cars/day and &Air pollution does not cause problems	1.05 (0.54–2.03)	1.06 (0.54–5.07)
Traffic > 10,000 cars/day and &Air pollution cause problems	2.12* (1.29–3.50)	2.14* (1.29–3.55)
Men poor health		
Traffic < 10,000 cars/day and &Air pollution does not cause problems	Referent group	Referent group
Traffic > 10,000 cars/day and &Air pollution does not cause problems	0.59 (0.20–1.80)	0.58 (0.18–1.82)
Traffic > 10,000 cars/day and &Air pollution cause problems	1.41 (0.69–2.89)	1.52 (0.73–3.19)
Women poor health		
Traffic < 10,000 cars/day and &Air pollution does not cause problems	Referent group	Referent group
Traffic > 10,000 cars/day and &Air pollution does not cause problems	1.64 (0.69–3.90)	1.77 (0.73–4.28)
Traffic > 10,000 cars/day and &Air pollution cause problems	3.19* (1.57–6.51)	3.21* (1.55–6.65)

*p < 0.05; OR univariate odds ratios; ‡aOR adjusted odds ratios for: age, educational status, family status, situation at work, monthly net income, NDVI (continuous), and smoking status

## Discussion

### Demographic risk factors for poor self-rated health

This environmental epidemiological study increased societal awareness about the links between residential environment quality and gender health showing that the poor self-rated health is a result of multiple factors residential environment quality and gender health showing that the poor self-rated health. This is a result of multiple factors. This is one of the first epidemiological study in an Eastern European country investigating environmental impact on the disparities in gender poor health risk in an Eastern European country. Investigating environmental impact on the disparities in gender poor health risk. Our tested hypothesis that the built and social environment may have different impact on men’s and women’s poor self-rated health, was partly confirmed.

Seeking to estimate the risk factors for poor-health, we analyze whether the individual-level and environmental-level factors were associated with poor self-rated health in men and women. Then, the factors associated with poor health, were included in the multivariate logistic regression models. The analysis revealed that demographic factors (age, situation at work and income), environmental factors (perceived air pollution in the place of residence and irregular visits to green space), and personal factors (physical activity and chronical diseases) had different impact on the magnitude of poor health risk in men and in women. This study findings presented evidence that participants with lower SES, poorer residential neighborhood, irregular visits to green space and chronic diseases more often reported poor health. Perceived air pollution, irregular visits to green space and chronic diseases were consistently associated with poor health risk in men and women, yet part time job and low income had a higher impact on women’s poor self-rated health. These factors are treated as a risk factors for poor self-rated health.

The findings of this study are partially consistent with the results of the studies presenting that the SES may impact disparities in human health and well-being [35, 61–63]. Like in this study, suggested possible determinants in subjective health disparities are behavioral factors (smoking, low physical activity), the psychosocial environment [22, 23], the burden of disease [64], and multi-morbidity [65]. There is a suggestion that the observed inequality between the genders in cross-country differences depends on role-related social norms, leisure time activity [66], and personal safety in urban spaces [67]. Thus, women may have more safety-related problems to visiting public spaces, such as parks, leading to adverse health outcomes. Our study did not confirm such circumstances but showed that situation at work have the highest impact on the association between environmental factors and poor health in women. Women were better educated than men were, but their economic indicators of the position at work and income were worse. Similar findings presented other recent studies [68, 69].

In this study, among men and women direct association between age group and poor health status was evident. The findings did not confirm significant differences in the prevalence of poor self-rated health in 18–75-year-old sample of men and women. Among the men and women aged 65 years and over the prevalence of poor health was 43.1%. Significant differences in the risk of poor health among men and women was found in 45–64-year-old group (aOR 4.73 (2.19–10.21) and aOR 2.65 (1.42–4.94), respectively). The findings presented evidence that age is among the risk factors that significantly increase prevalence of poor self-rated health in men and women. These findings are consistent with the results previously study’s conclusions that general poor self-rated health increase with age and age is treated as predictors of poor self-rated health [70–72].

### Impact of environmental and demographic factors on women’s and men’s poor self-rated health

Previous studies have demonstrated different health effects estimates of environmental exposure for males and females. This study results demonstrate that perceived air pollution in the residence place and irregular visits to green space were the risk factors consistently associated with the poor self-rated health in men and in women. Comparison of men and women data revealed that similarities and some differences exist between the men and women perceptions of neighborhood quality and social wellbeing. Women are more concerned about air pollution in their place of residence causing health problems, and higher than men rated the public transport in the district, indicating that it met their needs. Air pollution had significantly higher impact on women’s poor health. There is good scientific evidence that regular visits to green spaces can improve health, and green space near the home may be beneficial for physical and mental health.

This study findings show that some the participants’ environmental concerns and neighborhood quality rating scores differs between men and women. Both men and women were satisfied with district infrastructure and the possibility for walking to reach the city’s green spaces or parks. High levels of satisfaction with the participants’ neighborhood infrastructure and safety create the possibility for physical activity in green spaces. However, the physical activity was poor among both men and women: only 56.9% men and 41.1% women regularly visited the natural environment, yet 84.5% men and 85.7% women did not reach the recommended limits. The previous studies whose investigated associations between availability of green spaces and greenness-based physical activity did find different outcomes: positive associations [73, 74], uncertain [75] or heterogeneous results [43, 76, 77]. Some studies suggests that the residential proximity to green spaces did not influence general health, however, the usage of green spaces differs between men and women, claiming that men more frequently use green spaces and are more physically active in green spaces [78]. The presence of such mixt research results leads to the postulation that neighborhood infrastructure facilitating accessibility and physical activity in green spaces alone does not necessary increase people physical activity. Supporting information about the health benefits of the visits to green space and physical activity must be available to citizens.

In this study, neighborhood quality in the place of residence (traffic flow higher than 10,000 cars/day and air pollution cause problems) had a greater effect on health in women than in men, while a limited contact with the natural environment increases health problems in both men and women. Therefore, regular visit to green space may be beneficial for health. These our data are consistent with the results of the studies presenting that urban green spaces are associated with better general health and physical activity in green environment [79, 80], and that improving the neighbourhood environment would promote increased physical activity, such as reaching green spaces by walking, might contribute to the well-being of urban residents [81, 82].

Our findings indicates that the risk of poor self-rated health in men and women is outcome of low physical activity. There is good scientific evidence that reaching the recommended physical activity levels in green spaces would help people stay at a healthy weight. This conclusion conform to the findings reported in other studies [20, 26] showing that higher physical activity might reduce the risk of chronic diseases and have positive impact on general citizens health.

In this study, information collected was relevant to SDG 5 Gender Equality indicator and include of the number or percentages of both health and SES indicators. The study presented educational attainment, situation at work, monthly net income, gender chronic disease and self-rated poor health. The main factors affecting the size of the gender gap in self-rated poor health were the female-male gaps in the prevalence of chronic disease and gender situation at work. The findings showed that gender per se was not a risk factor for poor self-rated health. Some studies have also found no gender differences in poor self-rated health [18]. There are some data indicating that in different European countries the gender inequalities of health are explained largely by social conditions, particularly wealth [25] and medical conditions [65]. Our findings show that urban built and social environment, individual-level factors, chronical diseases, and health behavior are the risk factors that have joint outcome on health in men and women. Thus, it is evident from these results, that inconsistency in the reported poor health prevalence between men and women in different studies many depend on unequal prevalence of the factors, which can increase the likelihood of developing a chronic disease and the risk of poor general health.

### Strengths and limitations of the study

The strengths of this study, in relation to other studies, include a large sample size, the usage of formalized questionnaires for measure of the environmental quality perception and multivariate analysis. These measures helped to gain new knowledge on men and women differences in environmental-level concerns and self-rated health. The usage GIS for join environmental exposures and personal-level factors allowed us to investigate specific associations and gender differences. Moreover, in logistic regression models, we controlled the studied associations for the possible confounding variables and presented evidence on the effects of the built and social environment on poor self-rated health in men and women. The findings revealed that urban built and social environment and individual-level factors had a joint effect on the prevalence of poor self-rated health in men and women. SES, air pollution, and low physical activity in green space had a higher impact on women’s poor self-rated health. Our results suggest that decreasing air pollution and improving the urban built neighborhood supporting citizens’ physical activity in green space, might reduce health risks for all.

However, there are some limitations that should be acknowledged. We conducted a cross-sectional study which describes the strength of existing associations but is limited evidence about the causation of health problems. Nevertheless, during multivariable analysis controlled for possible confounding variables, some non-reported behavioral, socio-demographic, or perceived environmental variables are possible. Subjectively estimated physical activity – i.e., by using a questionnaire should be considered with caution. The traffic-related environmental exposures were presented at the district level and may have an impact on misrepresented exposure indicators. We also did not analyze meteorological data, which may influence leisure time outdoor physical activity and could have confounded our results. However, the changes to traffic-related exposures in their place of residence and physical activity did not depend on gender. The usage of qualitative variables in multivariate logistic regression is a potential limitation that may have biased the findings. In future studies, objective environmental quality and physical activity measurements using sensors would provide higher validity data. Advancing health equity and gender concepts in environmental health studies comprising an individual level, reasonable theoretical foundation, and accounting mechanisms of privilege and disadvantage of gender might present evidence-based data for public health [4, 83, 84].

Our findings suggest that efforts to reduce poor health among men and women may benefit from improving the physical and social environment that improve the neighborhood walking environment. People are more likely to make healthy behavior choices when these choices are easily available to them. The study results highlight the complex relationship between environmental issues, physical activity, health, and gender. These domains interact with the sustainable development goals across social determinants, health behaviors and health [45].

## Conclusions

The results of this environmental epidemiological study provide evidence that the quality of the built neighborhood and social environment, individual-level characteristics, chronical diseases, and irregular visits to green space were the factors that influenced a higher prevalence of poor self-rated health in men and women. Perceived air pollution in the place of residence and low physical activity in green space have a higher impact on women’s poor self-rated health than men’s have and are important determinants of poor health. However, women gender per se was not a determinant of poor self-rated health. Our work has implications for sustainable cities and society by suggesting that improving the physical and social environment of the neighborhood, ecological design, creation the opportunities for walking to reach the city’s green spaces or parks might benefit the men and women health and well-being. Measures oriented towards physical activity in green space should be encouraged among citizens to decrease the risk of chronical disease and poor health.

## Supplementary Information

**Additional file 1.**

## Data Availability

All data generated and analyzed during this study are presented in the main manuscript.
