# Supplementary material for: Environmentally related gender health risks: findings from citizen science cross-sectional study
Source: BMC Public Health. 2022 Jul 27;22:1426. doi: 10.1186/s12889-022-13824-3 (PMC9325661; doi:10.1186/s12889-022-13824-3)
Supplement: Supplementary file 1 — Additional file 1. [file 12889_2022_13824_MOESM1_ESM.docx]

**Environmentally related gender health risks: findings from citizen science cross-sectional study**

**Table S1** Descriptive statistics for men and women

| **Gender**  **Characteristics** | **Men**  **N (%)** | **Women**  **N (%)** | ***p*** |
| --- | --- | --- | --- |
| Age groups |  |  | *< 0.003‡* |
| 18–44 | 173 (34.7) | 187 (31.8) |  |
| 45–64 | 311 (62.4) | 357 (60.7) |  |
| >= 65 | 14 (2.8) | 44 (7.5) |  |
| Family status |  |  | *0.218‡* |
| Married | 296 (59.4) | 327 (55.6) |  |
| Other | 202 (40.6) | 261 (44.4) |  |
| Educational status |  |  | *0.100‡* |
| Lower | 247 (49.6) | 262 (44.6) |  |
| University | 251 (50.4) | 326 (55.4) |  |
| Situation at work |  |  | *< 0.001‡* |
| Full-time | 368 (74.0) | 361(61.5) |  |
| Part-time | 129 (26.0) | 226 (38.5) |  |
| Monthly net income |  |  | *0.030‡* |
| Less than 400 € | 66 (13.3) | 107 (18.2) |  |
| More than 400 € | 432 (86.7) | 481 (81.8) |  |
| Smoking |  |  | *< 0.001‡* |
| No | 349 (70.1) | 461 (78.9) |  |
| Yes | 149 (29.9) | 123 (21.1) |  |
| Recommended physical activity | |  | *0.608‡* |
| <150 min/week | 421 (84.5) | 504 (85.7) |  |
| >=150 min/week | 77 (15.5) | 84 (14.3) |  |
| Body mass index (BMI), (mean (SE)) | 26.14 (0.176) | 24.72 (0.196) | *< 0.001†* |
| BMI(<30) | 431 (87.4) | 508 (87.9) | *0.852‡* |
| Obesity (>=30) | 62 (12.6) | 70 (12.1) |  |
| Systolic blood pressure, (mean (SE)) | 125.46 (0.647) | 124.37 (0.617) | *0.230†* |
| Diastolic blood pressure, (mean (SE)) | 84.72 (0.460) | 82.01 (0.451) | *< 0.001†* |
| Chronic disease |  |  | *0.324‡* |
| No | 351 (70.5) | 398 (67.7) |  |
| Yes | 147 (29.5) | 190 (32.3) |  |
| Hypertension |  |  |  |
| No | 353 (70.9) | 423 (71.9) | *0.736‡* |
| Yes | 145 (29.1) | 165 (28.1) |  |
| Health status |  |  | *0.311‡* |
| Good | 427 (85.9) | 491 (83.5) |  |
| Poor | 70 (14.1) | 97 (16.5) |  |
| Traffic 10,000 cars/day |  |  | *0.686‡* |
| No | 352 (71.0) | 424 (72.1) |  |
| Yes | 144 (29.0) | 164 (27.9) |  |
| Noise (dBA mean (SE)) | 48.57 (0.18) | 48.44 (0.17) | *0.614†* |
| NO2 (μg/m3 mean (SE)) | 16.55 (0.07) | 16.50 (0.07) | *0.678†* |
| PM2.5 (μg/m3 mean (SE)) | 19.54 (0.04) | 19.51 (0.04) | *0.640†* |
| PM10 (μg/m3 mean (SE)) | 27.84 (0.05) | 27.80 (0.05) | *0.622†* |
| NVDI (mean (SE)) | 0.30 (0.00) | 0.31 (0.00) | *0.560†* |

† p value of Student’s t test; ‡ p value of the chi-squared test; SE – standard error

**Table S2** Univariate and multivariate associations between self-rated poor health status and age groups in men and women

| **Age groups, years** | **Self-rated health** | | | |
| --- | --- | --- | --- | --- |
|  | **Good health, N (%)** | **Poor health,**  **N (%)** | **Univariate,**  **OR (95% CI)** | **Adjusted†,**  **aOR (95% CI)** |
| Men and women |  |  |  |  |
| 18–44 | 337 (93.6) | 23 (6.4) | 1 (referent) | 1(referent) |
| 45–64 | 548 (82.2) | 119 (17.8) | 3.18* (1.99–5.07) | 3.30* (2.04–5.32) |
| >= 65 | 33 (56.9) | 25 (43.1) | 11.10* (5.68–21.69) | 9.65* (4.70–19.80) |
| Men |  |  |  |  |
| 18–44 | 164 (94.8) | 9 (5.2) | 1(referent) | 1(referent) |
| 45–64 | 253 (81.6) | 57 (18.4) | 4.11* (1.98–8.52) | 4.73* (2.19–10.21) |
| >= 65 | 10 (71.4) | 4 (28.6) | 7.29* (1.91–27.83) | 8.98* (2.15–37.40) |
| Women |  |  |  |  |
| 18–44 | 173 (92.5) | 14 (7.5) | 1(referent) | 1(referent) |
| 45–64 | 295 (82.6) | 62 (17.4) | 2.60* (1.41–4.78) | 2.65* (1.42–4.94) |
| >= 65 | 23 (52.3) | 21 (47.7) | 11.28* (5.05–25.21) | 8.70* (3.67–20.63) |
| Age group 18–75 |  |  |  |  |
| Men | 427 (85.9) | 70 (14.1) | 1 (referent) | 1 (referent) |
| Women | 491 (83.5) | 97 (16.5) | 1.21 (0.86–1.68) | 1.05 (0.74–1.49) |

*p<0.05; OR, odds ratios; †aOR, adjusted for: educational status, family status, situation at work, monthly net income, NDVI, and smoking status
